# Caecal Epiploic Appendagitis Masquerading Clinically as an Acute Appendicitis: A Case Report and Brief Literature Review

**DOI:** 10.1155/2019/6508642

**Published:** 2019-01-15

**Authors:** Tallat Ejaz, Eltaib Saad, Andik Nabil, James Slattery

**Affiliations:** ^1^Department of Surgery, Midland Regional Hospital Mullingar, Longford Road, Mullingar, Co-Westmeath, Ireland; ^2^Department of Radiology, Midland Regional Hospital Mullingar, Longford Road, Mullingar, Co-Westmeath, Ireland

## Abstract

A 46-year-old female presented to our emergency department (ED) with a 2-day history of right lower abdominal pain which was associated with nausea and anorexia. Abdominal examination revealed tenderness in the right iliac fossa (RIF) with rebound tenderness and a localized guarding. Urine dipstick was normal, and the pregnancy test was negative. Her laboratory investigations were significant only for a CRP of 16.6. A presumptive clinical diagnosis of acute appendicitis was suggested based on the given history and relevant physical signs. However, an abdominal computed tomography (CT) scan revealed an epiploic appendagitis of the caecum with a normal-looking appendix. She was managed conservatively and responded well and was discharged after 2 days in good health. Though being a relatively rare case of acute localized right-sided lower abdominal pain, caecal epiploic appendagitis should be considered as one of the differential diagnoses with the final diagnosis reached usually by the radiological findings due to the nonspecific nature of clinical and laboratory features.

## 1. Background

Epiploic appendages are pedunculated, fat-filled peritoneal pouches supplied by small blood vessels that form vascular stalks that contain branches of a circular end-artery and a central draining vein that supply the corresponding segment of the colon [1, 2]. They are found spread all over the serosal surface of the colon, but they are much more abundant and larger on the sigmoid and transverse colon walls [1, 2].

Surgical events arising from epiploic appendages are a fairly rare occurrence, and these include inflammation, torsion, internal herniation and obstruction, and colonic intussusception [2–4]. Epiploic appendagitis is an ischemic infarction of an epiploic appendage caused by torsion or thrombosis of the central draining vein. In fact, it is an unusual presentation and often a misdiagnosed cause of acute localized abdominal pain mimicking other relatively common entities such as left- or right-sided acute diverticulitis, acute appendicitis, acute cholecystitis, and possibly omental fat infarction and thus causing a real diagnostic dilemma [2–6]. This would arguably explain its importance as a possible, though rare, differential diagnosis for the acute localized abdominal pain. The misdiagnosis would lead to unnecessary hospitalizations and surgical intervention as the condition is benign and typically self-limiting with a favorable long-term outlook [1, 3, 6]. Herein, we present a case of a 46-year-old female patient with a diagnosis of caecal epiploic appendagitis which was initially thought as a case of an acute appendicitis based on the typical history and physical findings of the latter.

## 2. Case Presentation

**Figure 1:**
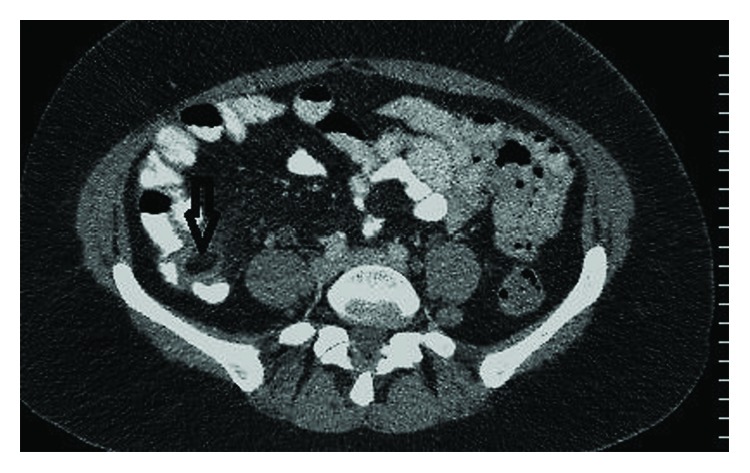
Axial CT abdomen with oral contrast showing caecal epiploic appendagitis with minor pericaecal fat stranding (vertical white arrow).

**Figure 2:**
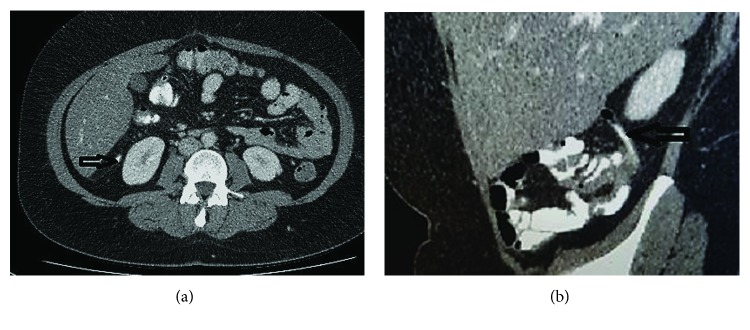
(a) Axial CT abdomen image and (b) sagittal image. The appendix looked entirely normal (horizontal arrows in (a) and (b) images).

A 46-year-old woman presented to the emergency department with a 2-day history of right-sided lower abdominal pain. The pain started suddenly around the central abdomen and then moved towards the right side. It was worse with movement and was associated with nausea and anorexia. There was no vomiting, diarrhea, or rectal bleeding. She had normal bowel movements. No history of urinary or gynecological symptoms elicited. She had no previous similar presentations. Her past medical history was significant for sarcoidosis and recurrent respiratory tract infections. Generally, she looked unwell. She was afebrile. Vital signs on presentation were a pulse rate of 76 beats per minute, a blood pressure of 110/70 mmHg, and a respiratory rate of 14 breaths per minute. Systemic examination was essentially normal. Examination of the abdomen revealed marked tenderness in the RIF with rebound tenderness and a localized guarding. The rest of the abdomen was soft and nontender with normal bowel sounds. Blood tests revealed a WCC of 7.1 and a CRP of 16.6. Renal and liver function tests were within the normal ranges. Urinalysis was normal. The pregnancy test was negative. Based on the given history and relevant physical and laboratory findings, a presumptive clinical diagnosis of acute appendicitis was suggested. The patient was admitted for observation. A computed tomography (CT) scan of the abdomen and pelvis was performed the next morning, which revealed an epiploic appendagitis of the caecum with a mild surrounding pericaecal fat stranding, no collection or free air noted (Figure 1). The appendix looked entirely normal (Figures 2(a) and 2(b)). She was managed conservatively with analgesia and antibiotics for 2 days and made a complete recovery and was sent home. In a follow-up visit after a week, she was generally well and reported no recurrence of her symptoms. She was finally discharged from the surgical care.

## 3. Discussion

Epiploic appendagitis is a relatively rare surgical occurrence, but the true incidence is not yet known. However, it has been reported in 2-7% of patients with a presumed clinical diagnosis of acute diverticulitis and 0.3-1% of those suspected of having acute appendicitis [6].

Owing to the nonspecific nature of presenting symptoms, physical signs, and laboratory findings, it is extremely difficult to make the correct diagnosis before radiological investigations, and hence, the vast majority of the cases are detected incidentally on the CT scans while investigating or ruling out other intra-abdominal pathologies [7]. Historically, the diagnosis has been made during exploratory laparotomy, but currently, the radiological investigations have become the main diagnostic tool for epiploic appendagitis [1].

Ultrasonography scan (USS) of the abdomen has some characteristic features to diagnose epiploic appendagitis [8]. Nevertheless, the CT scan is the gold standard of diagnosis [1]. Typical tomographic signs are 2-3 oval-shaped lesions with central hyperattenuation corresponding to a thrombosed draining vein, thickened peritoneal linings, and inflammatory changes within the surrounding fat [8]. It is important to note that the epiploic appendages are not usually seen on the CT scan slices if they are not inflamed [8]. Magnetic resonant imaging (MRI) findings generally appear to correspond to CT ones; however, they are not well-studied [9]. Furthermore, the use of MRI in the diagnosis of appendagitis is limited by its nonavailability in many emergency settings.

Identifying caecal epiploic appendagitis diagnosis would undoubtedly avoid unnecessary hospital admissions, dietary restriction, antibiotic use, and perhaps surgical intervention and general anesthetic risks [2, 3, 6], but due to its rarity, it should not be routinely sought in every patient with lower right-sided abdominal pain, especially when other clinical and laboratory findings clearly point to acute appendicitis or other gynecological causes of the RIF pain.

The management of epiploic appendagitis is generally conservative with nonsteroidal anti-inflammatory drugs to control pain [3]. Complete resolution usually occurs between 3 to 14 days [8]. The role of antibiotics has been controversial in the literature, but many reports showed no added benefit of antibiotic use [1, 7].

In conclusion, caecal epiploic appendagitis is a rare surgical occurrence which mimics acute appendicitis with the diagnosis usually established by the relevant radiological investigations due to its nonspecific clinical and laboratory features. It is a self-limiting disease which requires only a conservative management. Surgeons and radiologists should keep it in their mind to avoid unnecessary hospitalizations, antibiotic use, and perhaps surgical interventions. Nevertheless, more common and certainly serious pathological conditions that necessitate urgent surgical or medical interventions should always be considered on the top of the differential diagnosis and ruled out before considering the diagnosis of caecal epiploic appendagitis.

## Additional Points

*Learning Points*. Caecal epiploic appendagitis is a relatively rare cause of the RIF pain mimicking acute appendicitis and other acute gynecological causes. Diagnosis is usually made by radiological imaging, particularly a CT scan of the abdomen due to the nonspecific nature of its clinical features and laboratory findings. Management is generally conservative with a favorable long-term outcome.
